# Physical Fitness Profile of High-Level Female Portuguese Handball Players

**DOI:** 10.3390/ijerph20095751

**Published:** 2023-05-08

**Authors:** Manoel Rios, Ricardo J. Fernandes, Ricardo Cardoso, Ana Sofia Monteiro, Filipa Cardoso, Aléxia Fernandes, Gonçalo Silva, Pedro Fonseca, João Paulo Vilas-Boas, José António Silva

**Affiliations:** 1 Centre of Research, Education, Innovation and Intervention in Sport, Faculty of Sport, University of Porto, 4200-450 Porto, Portugal or manoel.rios@hotmail.com (M.R.); up201200394@up.pt (R.C.); up201406202@edu.fade.up.pt (A.S.M.); up201402398@edu.fade.up.pt (F.C.); up201402859@edu.fade.up.pt (A.F.); up201807261@edu.fade.up.pt (G.S.); jpvb@fade.up.pt (J.P.V.-B.); jasdps@fade.up.pt (J.A.S.); 2 Porto Biomechanics Laboratory, Faculty of Sport, University of Porto, 4200-450 Porto, Portugal; pedro.labiomep@fade.up.pt

**Keywords:** handball, anthropometry, force, oxygen uptake, oral hygiene and health

## Abstract

We characterized the physical and physiological profiles of high-level female Portuguese handball players and examined the relationships between their anthropometric characteristics, general motor performance and cardiopulmonary fitness. Twenty-four high-level female handball players with an average age of 23.6 ± 5.5 years, height of 173.6 ± 5.1 cm and body mass of 72.6 ± 9.1 kg volunteered to participate. A Pearson correlation test was used to assess the relationship between variables. Direct relationships were observed between the players’ height and arm span (r = 0.741), as well as between their squat jump and countermovement jump performances with regard to body mass (r = 0.448 and 0.496, respectively). The 9 m jump shot has a large relationship with the 7 m standing throw (r = 0.786) and between left hand dynamometry and body mass index (r = 0.595). The 30 m sprint has a relationship with the 7 m standing throw (r = −0.526) and the 9 m jump throw (r = −0.551). Oxygen uptake has a relationship with the players’ height (r = −0.482) and time limit (r = 0.513), while the fitness index has a relation to the players’ height (r = −0.488) and arm span (r = −0.422). Our results should be considered when using physical testing to plan optimal physical training regimens in elite team handball.

## 1. Introduction

Handball is one of the most popular team sports, played in two 30 min halves with a 15 min break [1]. It is a game played at high intensity due to its constant changes of direction, repeated accelerations, physical contact between players, jumps and shots, in which passive play is not allowed [2,3]. The players’ anthropometry and body composition can decisively play a major role during the game [4,5]. Additionally, due to game characteristics, a player’s specific position requires different body dimensions and high force and power levels to resist multiple body contacts or to have numerous intensive bursts of activity [6]. By considering all game aspects and maximizing handball players’ development, adequate training control for monitoring physical fitness is crucial to avoid the possible risk of fatigue and injury [3,7].

Consequently, physical performance should be well developed to match the game requirements, with muscular strength and specific skills requiring considerable development to become an elite player [8]. These abilities involve different body segments, and high levels of speed, agility, endurance and muscle strength are necessary to include all-body training for a proper physical performance [9]. The speed of these actions determines a successful team that can undermine the opponent with fast attacks and shots (and can also promote the sport’s evolution) [8]. Evaluations are being carried out mainly by using general tests that reflect the required physical fitness and the intermittent and intense handball actions [8]. On the other hand, evaluating handball players allows the modeling of their individual physical profiles and the assessment of their aptitude after injury [8].

The physiological response of handball players has also been examined, showing that, although low-intensity activities (standing still and walking) in, for example, female elite team handball constituted about 75% of mean effective playing time, the players demonstrated a mean relative workload of around 80% of maximal oxygen uptake during the periods of effective match-play [10]. Considering the abovementioned characteristics, a high level of physical fitness is required in handball [8]. In fact, since at least 90% of the energy released during a match is aerobically driven and that players run ~4–6 km at a mean intensity close to 80–90% of their maximal heart rate and maximal oxygen uptake, the importance of aerobic capacity should not be underestimated [11].

Achieving and maintaining higher physical and physiological conditions also require strategies to prevent and monitor health status. As a part of general health, well-being and quality of life, oral health status is an indisputable determinant to take into consideration in sports, particularly at the high standards required in elite performance [12,13]. Therefore, it is important to investigate the current oral health status of sport practitioners and to understand whether poor oral health conditions could affect their performance. Since it is not easy to have access to elite players, this work presents an excellent opportunity to have an overview of the physical, physiological and oral health profile of a high-level female handball team. The aims of the current study were (i) to characterize the physical and physiologic profile of female high-level handball players and (ii) to associate their anthropometric characteristics with general motor performance and cardiopulmonary variables. Complementarily, we aimed to evaluate the overall oral health status of the female high-level handball team.

## 2. Materials and Methods

### 2.1. Participants 

Twenty-four high level female handball players, who were healthy, had no previous injuries, competed at national and international level, and were currently playing for the Portuguese national team, voluntarily participated in the current study. All players were assessed in two different occasions over a one-week period (with a minimum of 24–48 h between tests). In the first session, anthropometric and motor performance general characterization was conducted. In the second session, players completed a square wave transition running test from rest to the velocity at maximal oxygen uptake until voluntary exhaustion. Verbal encouragement was given during the exercise protocol. Players were informed about the experimental procedures and possible risks involved and signed a consent form to participate. The experimental procedures were approved by the Ethics Committee (CEFADE282020), following the Declaration of Helsinki and the guidelines of the World Medical Association for research on humans. 

### 2.2. Experimental Methods and Procedures

#### 2.2.1. Anthropometric Assessment

Regarding the anthropometric traits, stature was measured using a Seca 206 stadiometer (Seca GmbH., Hamburg, Germany, precision to the nearest millimeter), arm span was assessed with an anthropometer and a sliding caliper (Siber-Hegner, GPM, Zurich, Switzerland) and body mass, body fat and fat-free mass were obtained using a bioimpedance scale (InBody 230, Biospace, Seoul, Republic of Korea). The anthropometric technical error of measurement was 0.8 cm for stature, 0.9 cm for arm span, 0.1 cm for hand breadth and length, and 0.9 and 0.3 kg for body mass and fat-free mass, respectively. All measurements followed the International Working Group on Kinanthropometry protocols [14].

#### 2.2.2. Functional and Motor Performance Assessment

The players general motor performance was assessed using the following functional tests:

(i) Lower-limbs explosive power was estimated by performing a squat and a countermovement jump on a Bertec 6090 force platform (Bertec Inc., Columbus, OH, USA) operating at 2000 Hz and a horizontal jump with upper limbs swing [15];
(ii) Abdominal muscular strength and endurance was estimated by the maximum number of sit-ups in 60 s [14];
(iii) Shooting velocity was performed at 7 (standing) and 9 (jumping) m from a standard handball goal, without opposition, and measured with a Stalker II radar gun (Stalker ATS II, Richardson, TX, USA) positioned on a tripod at 2 m from the back of the goal;
(iv) Static handgrip strength was calculated using a hand-held digital dynamometer (T.K.K.5401 Grip-D, Takei, Japan, accuracy of ±2.0 kg), and performing force in a standing position was calculated with the dominant and non-dominant upper limbs fully extended [16];
(v) Upper-limb explosive power was estimated by throwing a 3 kg medicine ball from chest height while in a seated position against a wall [14];
(vi) Running velocity was estimated by performing a 30 m sprint using a photoelectric cell system Speed Trap II (Brower timing systems LLC., Draper, UT, USA) with 2.3% CV [14].

Two trials were performed for sit-ups and sprints, and three trials were conducted for vertical jumps, with the best value used for posterior analysis [17]. Three trials for each goal shooting, three trials for each hand at the handgrip test and three medicine ball throw trials were performed, with the mean value accepted for data analysis. At least 2 min of rest was observed between trials for all assessments.

#### 2.2.3. Cardiopulmonary Assessment

After an individualized running warm-up (of ~5 min duration at low intensity), handball players performed a square wave transition running test on a treadmill with 0% grade (AMTI, Watertown, MA, USA) [18] from rest to the velocity at maximal oxygen uptake until voluntary exhaustion to assess the time limit at this exertion. The participants velocities were established using the individual velocity of the last stage of the yo-yo intermittent recovery test level 2 [10]. Respiratory and pulmonary gas exchange variables were measured breath by breath during treadmill running using a K5 telemetric portable gas analyzer (Cosmed, Rome, Italy). Heart rate was also registered continuously by a monitor belt (Cosmed ANT+) that telemetrically emitted the data to the K5 portable unit. Capillary blood sample of 5 μL (Lactate Pro2; Arkay, Inc, Kyoto, Japan) for lactate concentration [La^−^] analysis was collected from a fingertip at the third minute after running.

The collected respiratory and pulmonary gas exchange data were examined to exclude occasional errant breaths caused by swallowing, coughing or signal interruptions. Only data between oxygen uptake mean ± 3 standard deviations were included for analysis, being subsequently smoothed using a moving and time average of 3 breaths and 10 s, respectively [19,20]. Individual peak values of oxygen uptake, minute ventilation, respiratory frequency, tidal volume and heart rate were determined using the last 30 s of exercise. The fitness index (mLO_2_∙kg^−0.73^∙min^−1^) was also calculated [21]. 

#### 2.2.4. Oral Health Assessment

Oral health screening was undertaken by an experienced dentist. The examination was conducted via a visual oral inspection of molar relationship (in accordance with Angle class), malocclusions, number of decayed, filled and missing teeth and periodontal status (visible supragingival plaque and/or gingivitis clinical signs) [12]. The oral hygiene was qualitatively classified as very good, good, fair and poor considering the overall oral health status of each player, and the use of mouthguards during training or actual competition was also registered. The procedures were painless, minimally invasive and conducted by using gloves and disposable plastic mirrors. All players were instructed to control and maintain good oral behaviors and were also warned of possible negative impacts on their performance resulting from oral health problems. Since our oral health inspection was a gross assessment of the players oral status, they were all encouraged to visit a dentist to provide an accurate diagnosis and treatment. 

### 2.3. Statistical Analyses

Computations were completed using GraphPad Prism 6, with mean and standard deviation values for descriptive analysis being reported for all variables. The Levene and Shapiro–Wilk tests were used to assess data homogeneity and normality. A Pearson correlation test was used to assess the relationship between the players anthropometrics, general motor performance and physiological variables and was interpreted as small (0.1–0.3), medium (0.3–0.5) and large (0.5–1.0). Significance level was set at 5%.

## 3. Results

### 3.1. Players’ Characterization

The high-level handball players’ average physical characteristics were as follows: 23.6 ± 5.5 years of age, a height of 173.6 ± 5.1 cm, a seated height of 137 cm, an arm span of 176.0 ± 7.9 cm, a body mass of 72.6 ± 9.1 kg, a fat free mass of 58.1 ± 6.5 kg, a body fat of 20.2 ± 0.1% and a body mass index of 24.1 ± 2.6 kg∙m^−2^. From the 24 players, 5 (20% of the total sample) were left hand dominant.

### 3.2. Motor Performance Assessment

Table 1 displays the general motor performance tests results, with players evidencing similar vertical jump heights with the squat and countermovement jumps, a higher velocity throw with a jump and higher right hand force values regardless of their hand dominance (*p* < 0.05).

### 3.3. Cardiopulmonary Assessment

Table 2 lists the data regarding the physiological variables assessed during the square wave transition running test. The oxygen uptake, minute ventilation, respiratory frequency, tidal volume, heart rate, fitness index and peak blood lactate showed high values (as expected) during treadmill running.

### 3.4. Oral Health Assessment

**Figure 1:**
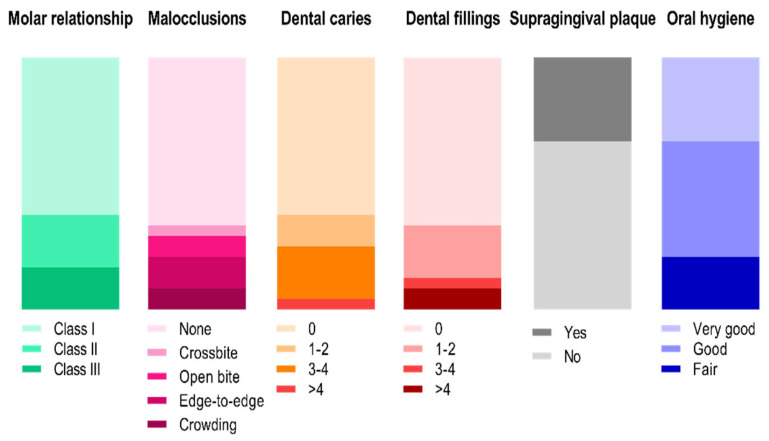
Oral health-related diagnosis observed for the handball players.

Data related to molar relationship, the presence of malocclusions, dental and periodontal health, and oral hygiene status are presented in Figure 1. Most players presented a normal molar occlusion (class I, 62.5%), and mesio and distocclusion were also observed (class II and III, 20.8 and 16.7%, respectively). Malocclusions were presented in the form of crossbite (*n* = 1), open bite (*n* = 2), edge-to-edge bite (*n* = 3) and dental crowding (*n* = 2). Overall, 15 players were free from decayed teeth, with the worst findings observed in 3, 5 and 1 players affected by 1–2, 3–4 and >4 dental caries, respectively. Meanwhile, 8 subjects presented at least 1 tooth filling, and, excluding third molars, only 2 players had missing teeth (tooth 36 and teeth 17, 36 and 37, respectively). An absence of supragingival plaque was verified for 16 players, and very good and good overall oral health hygiene were attributed to ~79% of the sample. The use of mouthguards during training or actual games was not reported by any player.

### 3.5. Relation between Variables

The relations between the assessed variables are displayed on Table 3. There is a large relationship between players’ height and their seated height and arm span, oxygen uptake and fitness index. A large relationship could also be seen between the horizontal jump and the countermovement and squat jumps, a medium relationship could be seen between the horizontal jump and seated height, and a small relationship could be seen between horizontal jump and body mass. The 9 m jump shot has a large relationship with the 7 m standing throw. Left hand dynamometry has a strong relationship with body mass, but right hand dynamometry has only a medium relation. The 10 m sprint has a medium relationship with the 7 m standing throw and the 9 m jump throw, while the 30 m sprint has a large relationship with the 7 m standing throw, 9 m jump throw and 10 m sprint. Oxygen uptake has a medium relationship with players’ height and a large relationship with time limit, while the fitness index has a medium relationship with players height, arm span and time limit and shows a large relationship with oxygen uptake.

## 4. Discussion

The aim of the current study was to characterize the physical and physiological profile of Portuguese high-level female handball players by relating their anthropometric characteristics, general motor performance and cardiopulmonary variables and by examining their general oral health status. Our results indicate that the female handball players had a good average body mass and good general motor performance [3,8]. In addition, the female handball players had an average maximal oxygen uptake like that of other female national handball teams [22]. As expected, the values for height, arm span and body mass are related to general motor performance and cardiopulmonary fitness, which are important factors for success in the game. Complementarily, and despite the fact that we have observed very good and good oral hygiene habits among our sample, malocclusions and poor oral health status were also verified, supporting the need for an improvement of dental status assessment in elite players.

In female handball, players’ physical characteristics are critical for obtaining success and are differentiated by appropriate morphological and motor features [23]. It was observed that the Portuguese female handball players values were shorter comparing to the Danish and Norwegian elite players but had a similar body mass [8,10]. In addition, our players presented a higher arm span and body mass index than female Spanish handballers [6]. Since handball is characterized by physical contact between players, the body dimensions have an important influence on their performance during the game since it affects the manifestation of motor abilities [3,24]. In fact, anthropometry affects the execution speed of specific handball technical elements, such as shots or defenses, and affects jump ability, which is a prerequisite for overcoming the opponent’s defense [25,26]. 

It has been showed that the game requires heavier players to not only block, push and pull the opponent but also to attack and shoot [27]. The current study showed that body mass was associated with countermovement and squat jump performances, highlighting that explosive actions depend on the capacity of the body to generate power. Meanwhile, the association between sprint values and the 7 m standing and 9 m jump throws suggests that higher sprint velocity did not result in higher values of ball velocity on throwing tests [27]. In addition, the positive association of ball velocity between the two throw types can be explained by the players higher muscle capacity to execute high speed movements and higher execution techniques [27]. Additionally, the higher ball velocity in the 9 m jump throw compared to the 7 m standing throw is due to the players’ three steps displacement before the throw, allowing an increase in ball velocity. It is known that grip strength is a determinant for catching, passing and throwing the ball, but hand dynamometry was not related to the throwing tests (in accordance with what was previously observed in other studies [27,28,29]). Lastly, trunk flexion and rotation, as well as shoulder internal rotation, were found to be the key factors in ball release speed [30]. 

The intermittent nature of the handball game enhances the need for a well-developed aerobic capacity that allows the players to maintain a high level of performance over the playing time [31]. In fact, it is well known that high aerobic performance improves the ability to tolerate high training intensity and volume, as well as recovery, especially during the more intense moments of the season when matches are played in a short period [10]. An interesting observation is the inverse relationship between height and oxygen uptake and between height and fitness index. In contrast with the literature [32], the taller players of the current study tend to have lower values of oxygen uptake and fitness index, providing important information regarding a potential weakness at their training level. The results of the assessed physiological variables demonstrated that the oxygen uptake of the Portuguese female handball players ranged between 34.7 and 59.9 mL∙kg^−1^∙min^−1^, with mean values similar to those of the Brazilian female national team [22] but lower than those of the Danish [33] and Norwegian female elite teams [10].

Complementarily, it is described that subjects engaged in sports, especially at professional and high-level standards, are more likely to develop poor oral health conditions. These includes dental caries, periodontal disease and wisdom tooth eruption, which enhance the risk of body inflammation, infection and muscle injuries, impacting overall health and the expected performance outcomes [34,35]. Regarding oral health-related problems, the current study data are from dental screening findings in national and international elite sport practitioners, reinforcing the need for oral health preventive/promotion programs [12,13,34]. We also found that the use of mouthguards to prevent the incidence of orofacial or dental trauma is still limited in our sample; however, since handball is a fast and high impact sport, strategies that emphasize mouthguard importance and mouthguard use during training and competition should be implemented [36]. Integrating oral health screening as a part of general health assessment and the use of effective promotion strategies are highly valuable for minimizing performance impacts from poor oral conditions. 

Our data allow the formation of training plans according to players’ individual and overall performance, which can particularly improve general motor performance and the physiologic characteristics that have a direct impact on the game outcome. In addition, data from the oral status are valuable as they help to prevent situations (e.g., acute pain or orofacial trauma) that could keep the players away from training or competition. A limitation of the current study was the lack of a larger sample that might have introduced selection bias in the results and that would allow for comparisons in terms of player seniority and position. Future studies should consider the influence of match efficiency. Since oral health assessments were conducted using simple dental tools and without specialized equipment (e.g., X-ray sources), our oral health-related data also need to be carefully interpreted because some oral diagnoses could be underestimated or not fully detected.

## 5. Conclusions

The current study provides valuable insights into the physical fitness profile of female handball players, particularly focusing on high-level Portuguese handballers. Our results highlight the importance of anthropometric characteristics related to general motor performance and cardiopulmonary fitness, which are crucial factors for success in the game and the general development of female players during the training season. In addition, oral health seems to be poorly monitored in the currently studied group, evidencing the need for oral health assessment in elite sports. Strategies to prevent oral disease and promote oral health need to be developed and evaluated in collaboration with a sports dentistry team.

## Figures and Tables

**Table 1:** Mean ± standard deviation values of players’ general motor performance variables.

Variables	*n* = 24
Countermovement jump height (m)	0.29 ± 0.03
Squat jump height (m)	0.28 ± 0.03
Horizontal jump (m)	1.9 ± 0.17
Medicine ball throw (m)	4.8 ± 0.41
Sit ups	40 ± 5
7 m standing throw (km/h)	80 ± 7
9 m jump throw (km/h)	83 ± 8
Right hand dynamometry (kgf)	38.4 ± 4.9
Left hand dynamometry (kgf)	34.9 ± 5.2
Sprint 10 m (s)	2.25 ± 0.25
Sprint 30 m (s)	5.04 ± 0.37

**Table 2:** Mean ± standard deviation values of players’ physiological variables.

Variables	*n* = 24
Time limit (s)	334.3 ± 161.2
Oxygen uptake (mL∙kg^−1^∙min^−1^)	46.5 ± 6.9
Minute ventilation (L∙min^−1^)	105 ± 13
Respiratory frequency (b∙min^−1^)	52 ± 9
Tidal volume (L)	2.1 ± 0.3
Heart rate (b∙min^−1^)	180 ± 8
Fitness index (mLO_2_∙kg^−0.73^∙min^−1^)	147.19 ± 18.82
Peak blood lactate (mmol∙L^−1^)	8.5 ± 2.5

**Table 3:** Pearson linear correlation for each variable.

	(1)	(2)	(3)	(4)	(5)	(6)	(7)	(8)	(9)	(10)	(11)	(12)	(13)	(14)	(15)	(16)	(17)
Height (1)	-																
Seated height (2)	0.556 **																
Arm span (3)	0.741 **	0.175															
Body mass (4)	0.353	0.410 *	0.203														
Body mass index (5)	−0.084	−0.049	0.153	0.132													
Countermovement jump (6)	0.330	0.479 *	−0.003	0.448 *	−0.360												
Squat jump (7)	0.289	0.394	−0.104	0.496 *	−0.337	0.890 **											
Horizontal jump (8)	0.025	0.330	−0.200	0.148	−0.232	0.576 **	0.483 *										
7 m standing throw (9)	−0.106	−0.162	−0.188	−0.190	0.049	−0.259	−0.106	−0.253									
9 m jump throw (10)	0.039	−0.036	−0.094	−0.199	−0.214	0.061	0.103	−0.250	0.786 **								
Right hand dynamometry (11)	0.248	0.355	0.341	0.448 *	0.395	0.055	0.066	−0.066	0.219	0.120							
Left hand dynamometry (12)	0.074	0.089	0.307	0.257	0.595 **	−0.132	−0.114	−0.043	0.034	−0.200	0.431 *						
Sprint 10 m (13)	−0.015	0.138	0.097	0.139	−0.056	0.179	0.004	0.097	−0.488 *	−0.456 *	0.042	−0.002					
Sprint 30 m (14)	−0.050	0.260	−0.063	0.170	0.082	0.079	−0.049	0.260	−0.526 **	−0.551 **	0.107	−0.013	0.833 **				
Time limit (15)	−0.083	0.066	0.045	0.199	0.249	−0.064	0.003	0.156	0.313	0.164	0.316	0.178	−0.049	0.016			
Oxygen uptake (16)	−0.482 *	−0.264	−0.385	−0.123	0.144	−0.266	−0.096	0.121	0.236	0.074	−0.073	0.196	−0.234	−0.051	0.513 *		
Fitness index (17)	−0.498 *	−0.215	−0.422 *	−0.124	0.107	−0.256	−0.094	0.156	0.257	0.075	−0.041	0.220	−0.202	−0.003	0.486 *	0.980 **	

** *p* < 0.01 and * *p* < 0.05.

## Data Availability

All of the data is contained within the article.
